# Results and prospects of development of new polyphenolic drugs for cancer patients

**DOI:** 10.18632/oncotarget.22307

**Published:** 2017-11-07

**Authors:** Vladimir N. Anisimov, Stig Larsen, Stig Lofberg, Irina A. Baldueva, Anastasia V. Malek, Tea Kirkegaard Nielsen, Elena I. Fedoros, Irina V. Perminova, Evgeny Yu. Drobyshev, Vladimir N. Bykov, Andrey V. Panchenko, Alexander M. Scherbakov, Alexey M. Belyaev

**Affiliations:** ^1^ N.N. Petrov National Medical Research Center of Oncology, Saint-Petersburg, Russia; ^2^ Norwegian University of Life Sciences, Oslo, Norway; ^3^ Meabco AS, Copenhagen, Denmark; ^4^ Oncosystema LTD, Moscow, Russia; ^5^ University of Copenhagen, Copenhagen, Denmark; ^6^ Nobel LTD, Saint-Petersburg, Russia; ^7^ M.V. Lomonosov Moscow State University, Moscow, Russia; ^8^ Institute of Toxicology, Federal Medical-Biological Agency, Saint-Petersburg, Russia; ^9^ S.M. Kirov Military Medical Academy, Saint-Petersburg, Russia

**Keywords:** cancer, breast cancer, clinical trial, polyphenols, cancer chemotherapy

## Abstract

The conference “Results and prospects of development of new polyphenolic drugs for cancer patients” took place at the N.N. Petrov National Medical Research Center of Oncology (PNMRCO) on May 31, 2017, and gathered researchers involved in development and evaluation of medicinal products based on the novel lignin-derived soluble polyphenolic polymer BP-Cx-1. BP-Cx-1 is the platform for a portfolio of innovative pharmacological products such as BP-C1, BP-C2 and BP-C3.

Natural substances, as safe and pluripotent candidates for designing medicinal products, draw interest of various scientific communities. At the present time methods for extraction and characterization of individual polyphenolic compositions and a solid evidence-based scientific database on potential molecular targets of these compositions have been developed.

Convincing nonclinical data, demonstrating, most of all, antioxidant properties of these compounds has been collected; some of these compositions (e.g., Genistein) are being evaluated in clinical trials.

At the same time, the data on pharmacological potential of derivatives of lignin, plant polyphenolic polymer are very scarce.

The conference “Results and prospects of development of new polyphenolic drugs for cancer patients” took place at the N.N. Petrov National Medical Research Center of Oncology (PNMRCO) on May 31, 2017, and gathered researchers involved in development and evaluation of medicinal products based on the novel lignin-derived soluble polyphenolic polymer BP-Cx-1. BP-Cx-1 is the platform for a portfolio of innovative pharmacological products such as BP-C1, BP-C2 and BP-C3.

BP-C1, a novel platinum-containing antineoplastic intramuscular drug developed for therapy of a number of cancer indications, is currently under evaluation in randomized clinical trials in patients with metastatic breast cancer. BP-C2, a novel radioprotector/radiomitigator, incorporating ammonium molybdate, is developed for supportive care of cancer patients treated with chemo/radiotherapy. BP-C3, a novel pharmaceutical composition, comprising iron, selenium, A and C vitamins, is in animal studies as a geroprotector and a supportive care product for cancer patients.

Over 150 international scientists from research centers of 6 countries have been taking active part in evaluation of potential of these products. To facilitate exchange of information and accelerate the translation from laboratory to clinical and commercial setting, a community of scientists with support from the pharmaceutical company Meabco AS (Copenhagen, Denmark) has arranged the scientific conference.

During the conference leading researchers presented results of their experimental work, inspiring panel discussions regarding the potential of this group of substances for the therapy and supportive care of cancer patients.

His complimentary address, Alexey M. Belyaev, Director of the N.N. Petrov National Medical Research Center of Oncology, devoted to the role of the PNMRCO in development of the new polyphenolic products and to the history of long-term productive collaboration between science and business.

Stig Loefberg, CEO of Meabco AS (Denmark), expressed his sincere gratitude to the management and scientific staff of the N.N. Petrov National Medical Research Center of Oncology for over a decade of productive collaboration, presenting data on the status of the project and sharing his vision on the role of products developed by the company in treatment and supportive care of cancer patients.

Stig Larsen (Norwegian University of Life Sciences, Oslo) presented data from two clinical trials of BP-C1 in metastatic breast cancer patients: «The effect of BP-C1 in treatment of Stage IV metastatic breast cancer (MBC)” and «The effect of BP-C1 on QoL and Toxicity in treatment of Stage IV metastatic breast cancer (MBC)”.

Two randomized, double-blind, placebo-controlled multicenter studies were performed with the similar semi-crossover design. Study I was performed in 6 centers in Russia and Thailand (N=30 patients) and study II was performed in 4 centers in Thailand (N=31 patients). The demographic characteristics of MBC patients were nearly equal in two studies except for BMI (28.4 kg/m^2^ in Study I vs 22.5 kg/m^2^ in Study II) and duration of disease (from the date of diagnosis: 6.25 years in Study I vs 4.5 years in Study II).

Patients allocated to either BP-C1 or equal looking placebo by block randomization for 32 days treatment. At the end of this period, the placebo patient were switched to active BP-C1 for additional 32 days of treatment. Patients who completed 32 days of BP-C1 treatment were offered the opportunity to continue on BP-C1 for an additional 32 days in an open-label extension protocol. After completion of the therapy with BP-C1 all patients were followed up for 28 days. The patients were given daily intramuscular injections of BP-C1 or placebo in the amount of 0.035 mg/kg of body weight for 32 days. Biochemistry, hematology, National Cancer Institute Common Terminology Criteria for Adverse Events (CTC-NCI), European Organization for Research and Treatment of Cancer quality of life questionnaire (QOL-C30 and the breast cancer-specific BR23) were recorded at screening and after every 16 days of treatment. Computed tomography was performed at screening and every 32 days. Response Evaluation Criteria In Solid Tumors (RECIST) was used for classification. The status of Estrogen receptor (ER), Progesterone receptor (PR) and Human epidermal epidermal growth factor receptor (HER2) prior to the study were collected after finalization of the studies.

The sum of target lesions diameters increased by 2.3% in Study I, and 8.9% in Study II in the BP-C1 group versus by 14.3% and 37.6% in the placebo groups after 32 days of treatment, respectively. The difference between the groups was significant in favor of BP-C1 (P=0.04, P <0.001). Additionally, a significant difference in favor of BP-C1 detected regarding the RECIST classification (P=0.026, P<0.001). Effect of BP-C1 was superior on MBC patients with double or triple negative ER, PR and HER2 receptors. The effect of BP-C1 increased with increasing number of negative hormone receptors.

No significant difference between BP-C1 groups and placebo groups was revealed regarding sum toxicity score (CTC-NCI) absolute change during 32 days of treatment in both studies (P=0.22). The increase in sum toxicity score was most pronounced in the placebo group.

“Breast cancer related pain and discomfort” scores (QLQ-BR23) were significantly reduced (P=0.02) in the BP-C1 group (-8.9 absolute change), but increased slightly in the placebo group (+6.5) in Study I after 32 days of treatment. In Study II, Breast cancer related pain and discomfort last week” score was unchanged in the BP-C1, but increased non-significantly (p=0.12) in the placebo group. The Between-group differences were significant in favor of BP-C1 (P=0.03, P=0.05).

In Study I, 26 AE related to the study drug was reported of which 20 recorded in the BP-C1 group and six in placebo. All the AE classified as mild to moderate. Among 20 AEs in BP-C1 group were nausea, blood lactate dehydrogenase increased and headache and among the six in Placebo group were proteinuria. In Study II, seven AE related to the study drug was reported. Of these, four in BP-C1 and three in Placebo. All the AE’s in the BP-C1 group classified as mild to moderate but one AE in the Placebo group classified as severe. No Serious Adverse Events (SAE) related to the treatment drugs reported in neither of the two studies.

In patients suffering from stage IV metastatic breast cancer, treatment with BP-C1 provided tumor growth arrest, was well tolerated, improved quality of life, and produced few and mild to moderate manageable AE’s. Antineoplastic effect of BP-C1 did not seem to depend on the hormonal status of the primary tumor.

Irina A. Baldueva (N.N. Petrov Research Institute of Oncology, Saint-Petersburg) devoted her presentation to results of analyses of cytokines in blood plasma of metastatic breast cancer patients from clinical trials of BP-C1.

Information about levels of cytokines in cancer patients is an important component of evaluation of immune status, required for understanding of the antitumor immune response. Increased levels of pro-inflammatory cytokines, such as IL-6 and TNF-alpha, are associated with chronic inflammations and frequently observed in cancer patients. Such chronic inflammatory status is further complicated through the use of chemotherapeutic drugs.

Analyses of cytokines of patients with metastatic breast cancer who took part in a clinical trial of BP-C1 was performed by the Scientific Department of Oncoimmunology of the PNMRCO. Sixty two samples of blood plasma collected from 25 female patients (at screening and after 32 days of interventions with BP-C1 or Placebo) with confirmed metastatic breast cancer were analyzed. 27 selected cytokines were measured with the Luminex xMpa, a multiplex assay method. The set of analyzed cytokines comprised pro-inflammatory and anti-inflammatory cytokines, as well as pleiotropic cytokines. ANCOVA test was applied to the results of the analyses (Meddoc, Norway).

It was shown that administration of BP-C1 did not affect levels of pro-inflammatory cytokines in cancer patients. Statistically significant changes in dynamics of IL-13 (p=0,028) and IL-7 (р=0,027) were noted in patients treated with BP-C1. IL13 – is a multifunctional interleukin responsible for pro- and anti-inflammatory reactions and regulation of haemopoiesis. IL-7 – lymphopoietic growth factor is mainly produced by non-hematopoietic cells: brain stromal cells, epithelial cells of the thymus and intestine, keratinocytes, liver cells, dendritic and follicular dendritic cells. Thus, detected induction of these cytokines in response to therapy with BP-C1 indicates positive effect of this agent on hematopoiesis.

Anastasia V. Malek (N.N. Petrov National Medical Research Center of Oncology, Saint-Petersburg; Oncosystema LTD, Moscow) delivered a talk on effect of BP-C1-based therapy on the profile of miRNA circulating in plasma of breast cancer patients. MicroRNAs (miRNAs), class of small regulatory RNAs, play essential role in cancer development and progression. Specific cancer-associated changes of the cellular miRNA profile have being demonstrated for various malignancies, including breast cancer.

Alterations of cellular as well as extracellular profiles of miRNAs are supposed to reflect various clinical characteristics: metastatic potency, drug resistance, total or disease-free survival. Evaluation of these alterations might have diagnostic and predictive value. Patients with metastatic breast cancer were randomized into two groups. Patients of the first group (N=11) were treated with BP-C1, patients of the second group (N=12) received a placebo treatment, prior to the cross-over to therapy with BP-C1. Each treatment course lasted 32 days, samples of the plasma were collected before onset of the therapy and after each treatment course. Thus, there were 2 samples from each patient of the first group, and 3 samples from each patient of the second group. Total RNA isolation and RT-qPCR analysis of 179 plasma/serum associated miRNAs were performed using reagents from Exiqon, Denmark. Results were normalized using complex references value and expression level of each miRNA was averaged over five groups of samples. Statistical significance of the observed expression shifts was evaluated using nonparametric Mann-Whitney U test (GraphPad Prizm 6.0).

It was established that BP-C1 therapy induces increase of plasma concentration of hsa-let-7e-5p, hsa-miR-532-5p, and decrease of plasma concentration of hsa-miR-26a-5p, hsa-miR-33a-5p, hsa-let-7b-5p, hsa-miR-30c-5p, hsa-miR-20a-5p, hsa-miR-122-5p, hsa-miR-223-3p, hsa-miR-126-3p, hsa-miR-18a-5p, hsa-miR-221-5p, hsa-miR-885-5p, hsa-miR-30e-3p. These changes of circulating miRNA profile had a concordant character in the two groups, while they were not observed in samples collected right after treatment with the placebo drug. Thus, in patients with metastatic breast cancer BP-C1 therapy induced specific changes in profile of circulating miRNAs. These changes might reflect efficacy/toxicity of the applied therapy and have a predictive value, which need to be further assessed.

Elena I. Fedoros (Nobel Ltd.; N.N. Petrov National Medical Research Center of Oncology, Saint-Petersburg) delivered a talk on results of simulation of biological activity of the novel polyphenolic ligand of BP-C family drugs (*in vitro* and *in silico*) addressing a complex problem of identification of biological targets for natural polymers. A combination of established experimental methods was proposed as a possible solution to this problem.

Biological activity of BP-Cx-1 - platform for the BP-C family drugs, was assessed by Dr. R. N. Karapetian and his group (ChemRar Institute, Moscow), using *in vitro* Cerep Diversity Profile (P9) screening panel. It was demonstrated that BP-Cx-1 interacts with 40 of the 97 tested targets.

Biological activity of BP-Cx-1 was also simulated *in silico*, using the following input parameters: results of HPLC/HPLC-MS, obtained by the scientific group headed by Dr. E. I. Savelyeva (Scientific-Research Institute of Human Hygiene, Professional pathologies and Ecology of Federal Medical-Biological Agency of Russia, Saint-Petersburg), results of CHN, 13C NMR and FTICR-MS, latter ones generated by the group of Prof. I. V. Perminova (M.V. Lomonosov Moscow State University, Moscow) and results of ^1^H NMR, generated by Dr. K. A. Krasnov (Institute of Toxicology, Saint-Petersburg). CHN analyses demonstrated that BP-Cx-1 does not comprise any nitrogen-containing components; NMR spectra indicate that BP-Cx-1 comprises predominantly highly substituted polyphenols. These findings were used as the basis for *in silico* screening.

To perform *in silico* biological activity screening in ChemBL chemical database, only components with molecular masses confirmed by both HPLC-MS and FTICR-MS methods, were selected. Matching biological activity of BP-Cx-1 identified in the *in vitro* and in the *in silico* test systems comprises 9 targets: Glucocorticoid receptor (NR3C1), Prostanoid EP2 receptor (PTGER2), Beta-2 adrenergic receptor (ADRB2), Vasopressin receptors (AVPR2, AVPR1A), Thyroid-stimulating hormone receptor (TRHR), Adenosine A1 Receptor (ADORA1), GABA trans-porter (SLC6A1).

These identified interactions suggest that BP-Cx-1 has its own pharmacological activity, first of all, effect on inflammations and stress.

Irina V. Perminova (M.V. Lomonosov Moscow State University, Department of Chemistry, Moscow) in her report entitled Distribution of BP-Cx-1 polyphenolic ligand tagged with [^3^H] in cells and tissues presented radiolabeling technique developed by Dr. G.A. Badun for preparing tritium-labelled polyphenolic BP-Cx-1 ligand and the data from cell distribution and pharmacokinetics studies conducted using radiolabeled BP-Cx-1. Advantages of using tritium label for pharmacokinetics studies include incorporation of its isotope – hydrogen – into BP-Cx-1 structure, long half-life - 12.323 ± 0.004 years, low energy of decay with a release of beta particle (E_mean_= 5.69 keV), and small length of beta-particle path in water (∼ 1 μm). The labeling was performed using tritium thermal activation technique, which provides for non-selective H/T exchange in polyphenolic ligand. BP-Cx-1 samples were dissolved at concentration of 5 mg/mL in 0.15 M NaCl and buffered with 10 mM Na_2_HPO_4_ to pH 7.0. The specific activities of BP-Cx-1 ligand were 8 Ci/g. Two cell lines were used for the studies - MCF7/R cells - human breast adenocarcinoma cells and NIH3T3 cells - normal mouse fibroblasts.

The studies on intracellular localization were conducted by Dr. N.S. Melik-Nubarov and his group using microautoradiography. The darkening of nuclear emulsion, which is indicative of label accumulation, was observed at the cell membrane and, which is of particular interest, in the nuclear and perinuclear regions of the cells. The higher number of “dark spots” covering cell nuclei was observed for tumor cells as compared to normal fibroblasts. The polyphenolic ligand (BP-Cx-1) showed high affinity for binding to the cell surface exceeding that of poloxamers. Specific feature of the tested samples is their ability to accumulate in the perinuclear region of both tumor and normal cells and in the nucleus. Much more pronounced nucleotropic effect was observed for the tumor cells as compared to the normal cells.

The labelled ligand (^3^H-BP-Cx-1) was also used for the pilot pharmacokinetic studies. In this experiment, BP-Cx-1 was administered at 80 mg/kg; 6 BALB/c mice were used: 3 mice were decapitated 5 min after and 3 mice – 30 min after intravenous injection of ^3^H-BP-Cx-1. Liquid Scintillation Counting was used to evaluate accumulation of [^3^H] label in organs of the experimental animals. It was shown that upon intravenous injection, the highest concentration was observed in lungs (1053 µg/g of organ weight at 5 min. and 1239 µg/g of organ at 30 min.). One order of magnitude lower concentration was detected in blood, heart, liver and kidneys. Two orders of magnitude lower concentration was detected in brain, muscles, pancreas, stomach and spleen. The same effect was observed when a sample of humic acid produced from coal was tested. Alternative experimental design and a different route of administration of the radiolabeled ligand (intramuscular instead of intravenous) need to be identified to improve understanding of the obtained results. The studies were conducted by Dr. G.A. Badun jointly with Dr. A.V. Panchenko (N.N. Petrov National Medical Research Center of Oncology, St. Petersburg) and Dr. Yu.V. Zhernov (Immunology Institute, Moscow).

Evgeny Yu. Drobyshev (N.N. Petrov National Medical Research Center of Oncology; Institute of Toxicology, Federal Medical-Biological Agency, Saint-Petersburg) in his presentation “Distribution of metals in animals after BP-C1 and BP-C2 treatment” described pharmacokinetics, toxicity and tissue distribution of the novel platinum-containing (BP-C1) and molybdenum-containing (BP-C2) products. Role of the polyphenolic ligands in transport and elimination of metals are actively discussed. Compared to the classic platinum antitumor agent – cisplatin, BP-C1, which also contains platinum, shows better platinum release kinetics and lower protein binding, which result in a lower nonspecific toxicity of this product. Besides, smaller concentration of platinum was detected in non-target tissues of animals treated with BP-C1. In case of BP-C2, which is a radiomitigative agent, effect of the polyphenolic ligand on molybdenum distribution pattern in tissues was detected. Abnormal accumulation of molybdenum in ovaries and unchanged tissue-to-blood ratio indicate that the mechanism of transport of this metal to ovaries is different from that to other tissues. At the same time, molybdenum concentration in the brain increased only 2 times, whereas based on our other molybdenum speciation studies, which demonstrated similar concentration of this element in serum and cerebrospinal fluid, we expected it to be the same. Based on this we conclude that molybdenum transport through blood-brain barrier is not unspecific. BP-Cx-1 significantly accelerated distribution and elimination speed of molybdenum administered with BP-C2.

Vladimir N. Bykov (S.M. Kirov Military Medical Academy, Saint-Petersburg) presented data on radioprotective and radiomitigative potential of novel polyphenolic compound. Drugs used for protection of human beings from ionizing radiation fall under several distinct groups. Radioprotectors (“chemical protection”) have short-term efficacy and act on physical-chemical and biochemical levels during absorption of the ionizing radiation energy. Radiomitigators – are agents with long-term efficacy, which promote accelerated recovery of radiosensitive tissues exposed to radiation. Radiomodulators (“biological protection”) – increase resistance of the body to radiation through modulation of biological processes and enhancement of antioxidant protective mechanisms.

Results of evaluation of radioprotective and radiomitigative potential of BP-C2 were presented. To evaluate efficacy of BP-C2 in Acute Radiation Syndrome (ARS) model with extended monitoring of hematology and hematopoietic system, small laboratory animals (CBA male mice and Wistar male rats) were subjected to ionizing gamma radiation in the doses from 4.0 to 8.0 Gy without bone marrow shielding. BP-C2 was administered by gavage daily once every second day (in total 10 times) at 3 dose levels: 6.0 to 156.2 mg/kg in mice and 3.0 to 93.7 mg/kg in rats. Total body irradiation was performed once after the 5th administration of the drug. After irradiation animals were monitored for 30 days.

BP-C2 increased survival of both mice and rats. The most pronounced radioprotective effect in mice was observed at dose of 81.0 mg/kg and in rats at dose 93.7 mg/kg. BP-C2 was most effective at radiation doses from 5.0 to 6.0 Gy.

In the control group, MLD (Median Lethal Dose) was estimated as 5.7 Gy in mice and 5.0 Gy in rats, while it reached 6.1 Gy both in mice and rats treated with optimal radioprotective doses of BP-C2. Dose Modifying Factor (DMF) of the drug constituted 1.1 in mice and 1.2 in rats.

It was found that BP-C2 at best protects from or at least improves the course of such clinical manifestations of the ARS as loss of weight, reduced consumption of food and water, signs of hypodynamia, including adynamia, dyspeptic symptoms and diarrhea, hemorrhagic syndrome, manifesting as sanguineous serous discharge from the eyes and nose.

The optimal dose of BP-C2 (81.0 mg/kg 10 times) increased weight of the spleen (56±4.0 vs. 27±2.0 in control group) and Colony-Forming Units count in the spleen (8.6±1.10 vs. 5.4±1.10 in Control group) in mice irradiated with 5.7 Gy.

In rats (5.0 Gy, 93.7 mg/kg of BP-C2 10 times), the myelokaryocyte count in the bone marrow was approximately 1.3 times higher that of the control group on day 3 post-exposure, and two-fold increase in the myelokaryocyte count was observed by day 14. Additionally, recovery of bone marrow hematopoiesis was shorter in the BP-C2 group compared to the control group.

Repeated administration of BP-C2 ensured dose dependent radioprotection against radiation in the median lethal dose range. The use of the drug seems to increase the likelihood of favorable resolution of ARS syndrome. BP-C2 accelerated bone marrow hematopoiesis recovery and exerted favorable effect on hematopoietic system.

Andrey V. Panchenko (N.N. Petrov National Medical Research Center of Oncology, Saint-Petersburg) in his report “Possibilities of using new polymeric polyphenol complex BP-C3 for the adjunct treatment of patients receiving chemotherapy” presented original data on the effects of BP-C3 on toxicity induced with 5-fluorouracyl in SHR mice and on efficacy of chemotherapy applied to mammary carcinoma bearing FVB/N mice transgenic with HER-2/neu.

Antitumor therapy using 5-fluorouracil (5-FU) and other cytostatic agents is often associated with various side effects, thus development of an adjunct treatment for amelioration of toxicity of chemotherapy in cancer is an important aim.

BP-C3 (80 mg/kg b.w. by gavage for 18 days) significantly reduced hematological and intestinal disorders associated with 5-FU (150 mg/kg b.w. single intraperitoneal injection). BP-C3, when administered on either preventative/therapeutic or therapeutic schedule, demonstrated different effects on hematological parameters of hematopoietic organs and peripheral blood. Administration of ВР-C3 24 hours after 5-FU ameliorated both leukopenia/myelosuppression and prevented anemia in the mice, while when BP-C3 was stared 7 days prior to 5-FU only improvement of white blood counts was achieved. Both administration schedules significantly improved intestinal crypt survival after 5-FU induced damage.

In another experimental setting, addition of BP-C3 to CAF-regimen, Gemcitabine or Gemcitabine with Cisplatin administered to animals with mammary tumors, was assessed. BP-C3 did not reduce efficacy of these regimens. Most efficient, in terms of antitumor activity, was CAF regimen, stabilizing tumor development in about 60% of experimental animals (p=0.0006 vs Control) and providing up to 64% reduction of tumor volume.

Thus BP-C3 has a potential as a supportive care product for cancer patients, where it could be used to ameliorate chemotherapy related toxicities without affecting tumor sensitivity.

Vladimir N. Anisimov (N.N. Petrov National Medical Research Center of Oncology, Saint-Petersburg) presented data from a study on evaluation of geroprotective and anti-carcinogenic effects of polyphenolic composition BP-C3 in female SHR mice. BP-C3 is a geroprotective composition developed on the basis of BP-Cx-1 polyphenolic ligand.

In this study, age-indicating parameters of 60 mice lifetime exposed to 0.005% solution of BP-C3 administered with drinking water (10 mg/kg daily) was compared to 60 mice of the control group.

Age-related dynamics of the body weight, food and water consumption were not affected by BP-C3 exposure in SHR mice. Treatment with BP-C3 decreased rectal temperature by 0.2-0.4 °C and postponed age-related switching-off of estrous function in the experimental animals. BP-C3 increased mean life span by 8.4% up to 646±18.7 days (p<0.05), mean life span of the last 10% surviving animals by 12.4% (to 863±3.7 days (p<0.01)) and life span of tumor-free mice by 11.6% (to 676±24.3 days (p<0.05)). A tendency in ability of BP-C3 to inhibit development of spontaneous tumors in SHR mice was detected (p=0.166, log rank test: age at detection of the first tumor increased by 16% and total number of detected tumors decreased 1.6 times. Tumors developed in both groups were predominantly mammary adenocarcinomas. Frequency of non-tumor pathologies did not differ statistically between the groups.

When compared with other known geroprotective compounds, such as metformin, rapamycin and melatonin with effects on energy homeostasis, reproductive homeostasis and adaptation (stress resistance, immune activity etc.), low toxicity of BP-C3, its effect on delay of aging of estrous function and on increased survival with delayed in tumor development strongly indicate geroprotective activity of this composition.

Tea Kirkegaard Nielsen (Meabco AS; University of Copenhagen, Copenhagen) in her report «Results from analyses of the immunoregulatory properties of BP-C family drugs» presented a proposition for the common action of the members of the BP-C family.

BP-C3 prolongs the lifespan of mice, BP-C1 shrinks tumors, and BP-C2 mitigates damages from radiation and improves tissue healing. The link between these activities is the immune system which plays a pivotal role for all phenomenons. The incidence of lifestyle diseases such as cancer, liver cirrhosis, obesity, diabetes, among others, are all concurrent with, or caused by, immune malfunctioning. The tissue surrounding or infiltrating a tumor (tumor microenvironment) is decisive for the growth rate and for the proliferation rate of the cancer cells. The tissue contains a myriad of inflammatory signals which are involved in activation of the immune system to attack the cancerous cells. However, if these cells also express immune suppressor signals, shielding them from attack, the inflammatory signals are much less harmful and can instead provide growth factors stimulating tumor growth.

Age-related susceptibility to opportunistic pathogens and lack of response to vaccines, are also linked to immune dysfunction, a phenomenon referred to as immunosenescence. Increased inflammatory milieu and unfavorable composition of immune elements are highlighted as the cause of immunosenescence. Obesity, alcohol consumption, exposure to pollution, and smoking, are all directly linked to increase in local or systemic inflammation. Skin damage is followed by local inflammation (damaged tissue and pathogen depletion) and secondarily, cell proliferation (tissue regeneration).

The BP-C family is a mixture of complex compounds constituting several aromatic carbohydrates rich in antioxidant moieties. Based on structure and medical characteristics we hypothesized that BP-C holds anti-inflammatory properties. To investigate this, we isolated CD14-positive cells (primarily monocytes) from healthy donors and exposed them *in vitro* to innate immune triggering factors (e.g. lipopolysaccharide, LPS) to stimulate inflammation. We observed that the inflammatory cytokine secretion from the monocytes were highly suppressed after incubation with BP-Cx-1. The results were intriguing; the inhibition applied not only to one cytokine but several, and these were the ones known to be most inflammatory (IFN-γ, TNF-α, IL-1b). We conclude that the BP-C family might cease excess of inflammatory elements and this tendency will be further investigated *in vivo*.

Conference was summarized by Alexander M. Scherbakov (N.N. Petrov National Medical Research Center of Oncology, St. Petersburg) who expressed sincere admiration of quality of the research results, presented by the speakers, emphasized strong potential that lies in development of new polyphenolic drugs for cancer patients, drugs that could nicely fit the trends observed in the modern oncology – extension of quality life of cancer patients.

